# Six Homeoproteins and a linc-RNA at the Fast MYH Locus Lock Fast Myofiber Terminal Phenotype

**DOI:** 10.1371/journal.pgen.1004386

**Published:** 2014-05-22

**Authors:** Iori Sakakibara, Marc Santolini, Arnaud Ferry, Vincent Hakim, Pascal Maire

**Affiliations:** 1 INSERM U1016, Institut Cochin, Paris, France; 2 CNRS UMR 8104, Paris, France; 3 Université Paris Descartes, Sorbonne Paris Cité, Paris, France; 4 Laboratoire de Physique Statistique, CNRS, Université P. et M. Curie, Université D. Diderot, École Normale Supérieure, Paris, France; 5 Université Pierre et Marie Curie-Paris 6, Sorbonne Universités, UMR S794, INSERM U974, CNRS UMR7215, Institut de Myologie, Paris, France; Univ. of Missouri, United States of America

## Abstract

Thousands of long intergenic non-coding RNAs (lincRNAs) are encoded by the mammalian genome. However, the function of most of these lincRNAs has not been identified *in vivo*. Here, we demonstrate a role for a novel lincRNA, *linc-MYH*, in adult fast-type myofiber specialization. Fast myosin heavy chain (*MYH*) genes and *linc-MYH* share a common enhancer, located in the fast *MYH* gene locus and regulated by Six1 homeoproteins. *linc-MYH* in nuclei of fast-type myofibers prevents slow-type and enhances fast-type gene expression. Functional fast-sarcomeric unit formation is achieved by the coordinate expression of fast *MYHs* and *linc-MYH*, under the control of a common Six-bound enhancer.

## Author Summary

Adult skeletal muscles are classified into fast-type and slow-type, which display different resistance to muscle atrophy and metabolic protection against obesity. We identify in this manuscript a new mechanism controlling *in vivo* adult muscle fiber-type specification implicating a long intergenic non-coding RNA, *linc-MYH*. We demonstrate a three-element genetic partnership, where an enhancer under the control of the myogenic homeoprotein Six1 functions as a regulatory hub to control fibre phenotype. In this partnership, the enhancer controls positively the expression of both the adjacent fast myosin heavy chain (MYH) gene cluster and of *linc-MYH. linc-MYH* is present only in adult fast type skeletal myofibers and controls their phenotype by suppressing slow-type gene expression. The regulation of *linc-MYH* could provide a lead for new therapeutic approaches or drug development.

## Introduction

Adult skeletal muscles are composed of slow and fast myofiber subtypes which selectively express the genes required for their specific contraction activity and metabolic properties [1]–[4]. These properties are acquired at the end of fetal development and during the neonatal period, when mixed skeletal myofibers expressing a panel of embryonic, fast and slow genes develop a specific slow or fast phenotype. The formation of efficient fast sarcomeric units, and Ca^++^ cycling and excitation/contraction/relaxation coupling in fast-myofibers, is achieved through the coordinate control of fast *Myhs* and associated fast sarcomeric genes (including *Tnnt3*, *Tnni2*, *Tnnc2*, *Atp2a1* and *Pvalb*) [2],[4]. Myofibers can be classified by their MYH expression profile: slow-type myofibers in mice express MYH7 (also known as MYHCI, β or slow), and fast-myofibers express MYH2 (MYHCIIA), MYH1 (MYHCIIX) or MYH4 (MYHCIIB). Fast *Myh* genes found in developmental and adult stages (*Myh3*, *Myh2*, *Myh1*, *Myh4*, *Myh8* and *Myh13*) are organized as a cluster within a 300 kb region on mouse chromosome 11 [5]. The spatio-temporal expression of one specific fast *Myh* gene at the *Myh* locus is reminiscent of the organization and expression of Globin genes at the beta-globin locus [6]. However, we are yet to investigate potential enhancers or the *Myh* locus control region (LCR) that could be responsible for sequential and specific *Myh* gene expression in myofibers. The coordination of fast-type and slow-type gene expression in fast myofibers is not currently understood. Distinct intramyofibrillary calcium transients, evoked by slow tonic motor neuron firing, induce a cascade of downstream signaling involving Calcineurin and CamK. This results in the activation of selective transcription activators and repressors in slow myofibres. However, the signaling pathways operating in distinct *Myh2*, *Myh1* and *Myh4* myofiber subtypes, which coordinate the activation of the other fast-type genes and the repression of slow-type genes, is less well understood [1]. Better knowledge of the mechanisms controlling muscle specialization and plasticity is important to enable the understanding and modulation of muscle adaptations in pathophysiological conditions.

Six homeoproteins are major myogenic transcription factors which directly bind to DNA sequences (called MEF3s) to control myogenesis [7],[8] and the genesis of fast-type myofibers during embryogenesis [9],[10]. In adult skeletal muscle, Six1 accumulates at a higher level in the nuclei of adult fast myofibers than in of slow myofibers. Forced expression of Six1 and its Eya1 cofactor in slow myofibers causes adult slow-twitch oxidative fibers toward a fast-twitch glycolytic phenotype [11]. Animals with a *Six1* KO present severe muscle hypoplasia and die at birth [12]. This prevents the *in vivo* analysis of the adult phenotype and the ability to investigate the direct or indirect involvement of Six1 in the spatio-temporal control of the expression of genes in the fast *Myh* cluster.

The mammalian genome encodes thousands of long intergenic non-coding RNAs (lincRNAs) which have multiple functions [13],[14]. Some accumulate in the cytoplam as miRNAs decoys [15],[16]. Others accumulate in the nucleus and participate to gene regulation through chromatin remodeling and epigenetic modifications [14],[17],[18]. Here, they may act as cis [19] or trans [20] transcriptional activators, as transcriptional repressors [21],[22] or through DNA-RNA triplex formation [23],[24].

In this study we identify a new lincRNA, *linc-MYH*, and the mechanism of its control of adult muscle fast fiber-type specification *in vivo*. We demonstrate a three-element genetic partnership, where an enhancer element under the control of the myogenic homeoprotein Six1 functions as a regulatory hub to control fiber phenotype. In this partnership, the enhancer positively controls the expression of both the adjacent fast *Myh* gene cluster and *linc-MYH*, suppressing slow-type gene expression and facilitating fast fiber-type specialization.

## Results

### Six1 binds directly to a newly identified enhancer of the *Myh* genes cluster

**Figure 1:**
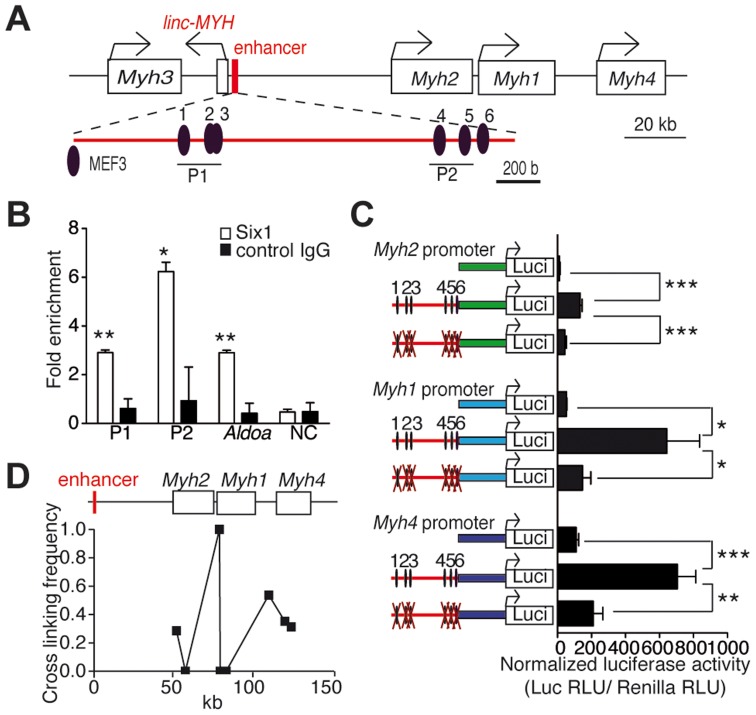
*Six1* binds directly the enhancer of the fast *Myh* gene cluster. (A) Schematic representation of the fast *Myh* gene cluster. (B) qPCR values of ChIP experiments performed with Six1 antibodies, or IgG on GP and TA chromatin, showing Six1 binding to P1 and P2 regions of the fast *Myh* enhancer, to the muscle promoter of *AldoA*, and to an intergenic region located 86 kb 5′ upstream of *Myh3* (NC) (n = 3). (C) Luciferase assays from adult TA muscles electroporated with luciferase vectors (indicated) and a TK-Rennila luciferase vector allowing normalization (n = 4). (D) qPCR experiments from 3C assays of wild type EDL muscle, showing the direct interactions of *Myh2*, *Myh1* and *Myh4* promoters with the fast *Myh* enhancer. *P<0.05, **P<0.01, ***P<0.001.

Our previous studies suggested that Six1 could be directly involved in the control of the expression of fast *Myh* genes, since higher levels of this transcription factor accumulate in the nuclei of adult fast myofibers than in slow myofibers [11]. To investigate how *Six1* could control the expression of fast *Myh* isoforms, we used computational analysis to locate MEF3 sites at the fast *Myh* locus (see Materials and Methods). Six clustered MEF3 sites are conserved across human, rat and mouse genomes in an intergenic region located 50 kb upstream of the *Myh2* gene (Figures 1A and S1) and 4 kb upstream of a lincRNA (2310065F04Rik); we refer to this lincRNA as *linc-MYH* (Figures 1A and S2). Six1 binding at these MEF3 sites was demonstrated *in vivo* by ChIP (Chromatin Immunoprecipitation) experiments with Six1 antibodies on adult fast gastrocnemius plantaris (GP) and tibialis anterior (TA) muscles (Figure 1B) but not on adult slow Soleus (data not shown), and confirmed for five of these sites (sites 1, 2, 3, 4 and 6) by EMSA assays (Figure S3A). We asked whether this *Myh* intergenic region could constitute an enhancer element, controlling the spatio-temporal expression of *Myh* genes in this locus. A 2 kb DNA fragment of this region, including the six identified MEF3 sites and 1 kb of DNA fragments upstream of the transcription start site of fast-type *Myh2*, *Myh1* and *Myh4* genes, was isolated. The putative enhancer was ligated to each *Myh* promoter using luciferase pGL3 basic plasmids to generate pGL3-Enhancer-*Myh2/1/4* constructs. To test the involvement of Six binding in enhancer activation of the *Myh2*, *Myh1* and *Myh4* promoters, we mutated all six MEF3 sites present in the enhancer, and named these reporters pGL3-mutEnhancer-*Myh2/1/4*. Luciferase activity was tested two weeks after the electroporation of reporter plasmids in adult TA muscles. The luciferase activity of pGL3-Enhancer-*Myh2/1/4* was seven to twelve times higher for either of the promoters, than with pGL3-*Myh2/1/4*. Enhancer activity was not observed in plasmids with mutated MEF3 sites associated with either of the *Myh* promoters (Figure 1C). Enhancer activity was neither observed with the promoters of the slow *Sln* (Figure S3B) or *Tnni1* genes, or with the promoter of the ubiquitous *β-actin* gene (Figure S3C). A weak enhancer activity was observed with *Myh4* promoter in primary embryonic fibroblasts, in which Six1 is expressed (Figure S3D and data not shown). These data showed that high MYH enhancer activity was only reached *in vivo* and required specific interactions with MYH promoter elements. To determine *in vivo* interactions between the enhancer and each *Myh* gene, we performed chromatin conformation capture (3C) assays of adult fast EDL (Extensor digitorum longus) myofibers. These experiments revealed that the enhancer interacts with the promoter of *Myh2/1/4* genes in native chromatin of EDL myonuclei (Figure 1D). The strongest interactions were observed with the *Myh1* and *Myh4* promoters, consistent with the expression profile of these two genes in EDL muscles. The data demonstrates that the identified conserved cis-element acts as an enhancer for the *Myh* locus and that MEF3 sites are essential for its enhancer activity *in vivo*.

### Loss of *Six1* impairs fast muscle genes and *linc-MYH* expression during post-natal development

**Figure 2:**
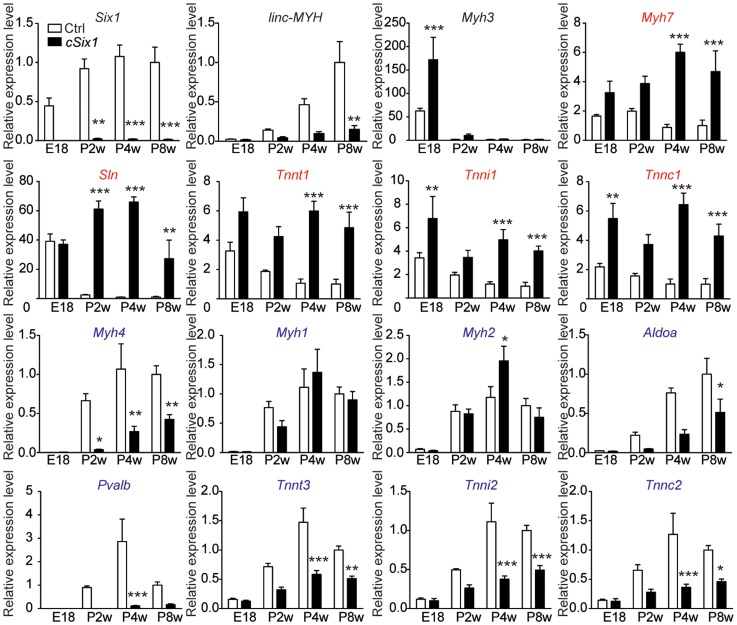
The expression of fast-type genes and *linc-MYH* is impaired in *cSix1 KO* mice during postnatal development. mRNA expression level of *Six1*, *linc-MYH*, *Myh3*, slow-type genes (red) and fast-type genes (blue) in back muscles of *cSix1 KO* mice at E18.5, P2W, P4W and P8W, as determined by qPCR experiments, (n = 3 to 6 for each point). *P<0.05, **P<0.01, ***P<0.001.

To further characterize the role of *Six1* in the control of fast *Myh* gene expression, we bred *Six1^flox/flox^* mice with transgenic mice expressing CRE recombinase under the control of the human skeletal actin (HSA) promoter and obtained *Six1^flox/flox^;HSA-CRE* conditional knockout mice (hereafter named *cSix1 KO*) [25],[26]. We analyzed the expression of fiber type specific genes in the back muscles of wild-type control mice and *cSix1 KO* mice at embryonic day 18.5 (E18.5) and at several post-natal stages (two weeks (P2W), four weeks (P4W) and eight weeks (P8W)) animals (Figure 2), as muscle fiber fast-subtype specialization takes place from the end of embryogenesis [9]. *Six1* mRNA was not detectable in back muscles of *cSix1 KO* mice (Figure 2). The expression of fast-type genes (*Myh4*, *Tnnt3*, *Tnni2*, *Tnnc2* and *Pvalb*) increased during postnatal development in control mice but that of slow-type genes (*Myh7*, *Tnnt1*, *Tnni1*, *Tnnc1* and *Sln*) decreased. The *linc-MYH* RNA was detected after birth in muscle samples and its expression increased in line with that of *Myh4* (Figure 2). The induction of fast-type genes and *linc-MYH* and the suppression of slow-type genes, were impaired in *cSix1 KO* mice. Expression of *linc-MYH* was reduced by three to five times in *cSix1 KO* mice during postnatal development (Figure 2). These results show that *Six1* controls the induction of *linc-MYH* and fast-type genes during postnatal development, and is required for the downregulation of slow type genes.

### *Six1* deficiency impairs adult muscle fast phenotype

**Figure 3:**
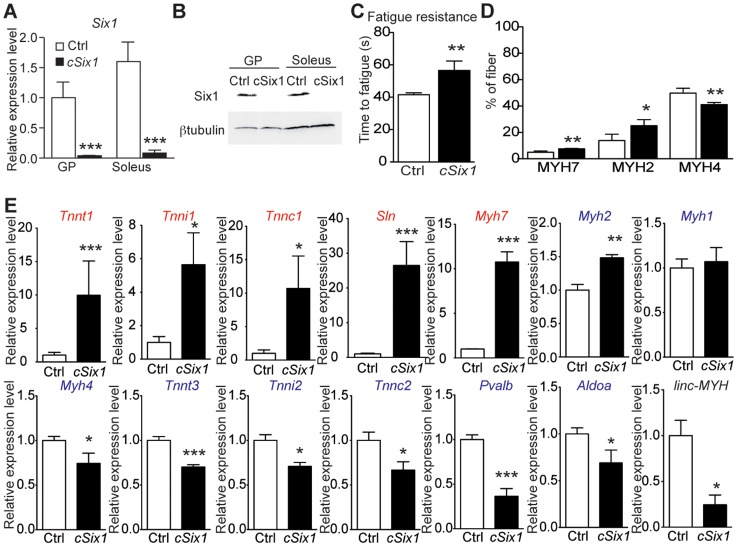
*Six1* deficiency impairs the adult phenotype of fast muscle. (A) *Six1* mRNA expression levels in GP and Sol muscles of three month-old control (Ctrl, n = 4) and *cSix1 KO* (n = 3) mice. (B) Western blot analysis of Six1 and βtubulin expression in Sol and GP of Ctrl and *cSix1 KO* mice. (C) Time to fatigue ratio of TA muscles of Ctrl (n = 4) and *cSix1 KO* (n = 4) mice. (D) Percentage of myofibers expressing MYH7, MYH2 and MYH4 in TA muscles of three month-old Ctrl (n = 4) and *cSix1 KO* (n = 4) mice. (E) mRNA expression levels of slow-type genes (red), fast-type genes (blue) and *linc-MYH* in TA muscles of three month-old Ctrl (n = 4) and *cSix1 KO* (n = 4) mice. *P<0.05, **P<0.01, ***P<0.001.

We next analyzed adult 12 week-old *cSix1 KO* mice to further characterize the role of *Six1* in adult muscle. *Six1* mRNA and protein were not detectable in GP enriched with fast-myofibers or soleus (SOL) muscle enriched with slow-myofibers (Figure 3A and B), and fatigue resistance of TA muscle was 35% higher (Figure 3C) in the *cSix1 KO* mice. We used immunohistochemistry to analyse the composition of MYH7, MYH2 and MYH4 in *cSix1* mutant myofibers. Mutant TA muscles had a higher percentage of fibers containing MYH7 and MYH2, but a lower percentage of fibers containing MYH4 (Figures 3D and S4). We found consistent results during qPCR analysis of *Myh* mRNA i.e., higher levels of *Myh*7 and *Myh*2 mRNA and lower levels of *Myh*4 mRNA levels were observed in the fast TA muscles of *cSix1 KO* (Figure 3E). Expression levels of other specific fast and slow-type genes were also tested. We found in mutant TA muscles a downregulation of fast-type genes (*Tnnt3*, *Tnni2*, *Tnnc2* and *Pvalb*) and a five and to 25 fold increase in the levels of slow-type genes (*Myh7*, *Tnnt1*, *Tnni1*, *Tnnc1* and *Sln*) (Figure 3E). Nevertheless expression of slow *Myh7* is increased more than ten fold at the mRNA level in *cSix1* mutant TA myofibers, while by immunohistochemistry the number of MYH7 positive myofibers is increased less than two fold. This showed that there is no major phenotype switch in *cSix1* mutant TA myofibers. This observation could be explained either by the higher amount of *Myh7* mRNA accumulating in MYH7 positive fibers or by a general increase of *Myh7* mRNA in TA myofibers, mRNAs that would not be translated efficiently and leading to the absence of increase of MYH7 positive fibers. The expression of *linc-MYH* expression was lower in the adult TA of *cSix1 KO* mice, than in control mice (Figure 3E). These results indicate that the *Six1* homeoprotein can control the phenotype of fast skeletal myofibers in adult animals.

### *linc-MYH* is expressed exclusively in adult fast-type muscles

**Figure 4:**
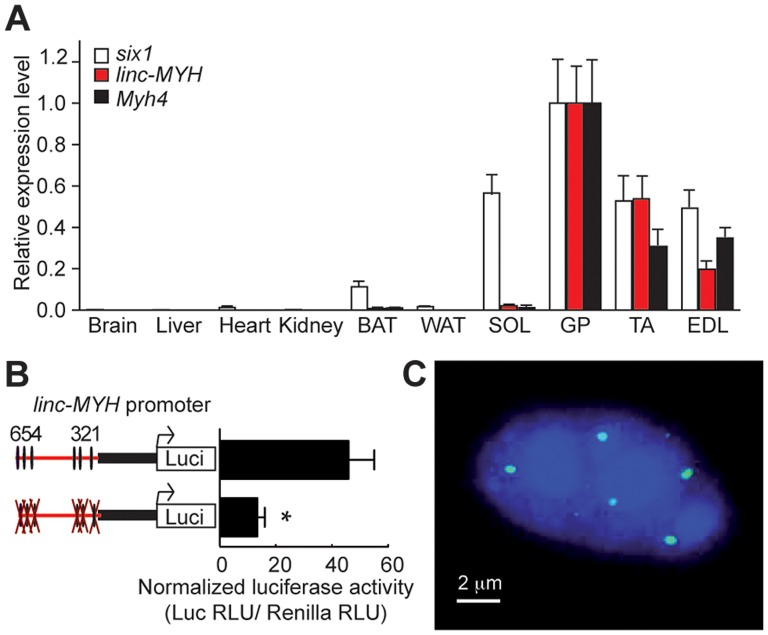
*linc-MYH* is expressed in adult fast-type skeletal muscles and accumulates in their nuclei. (A) Tissue distribution of *Six1*, *Myh4* and *linc-MYH* RNAs. BAT, brown adipose tissue; WAT, white adipose tissue. (n = 4). (B) Luciferase assays of adult TA electroporated with *linc-MYH* promoter luciferase vectors (indicated) and a TK-Renilla luciferase vector (allowing normalization). *P<0.05. (C) FISH of isolated EDL myofiber with a *linc-MYH* antisense RNA fluorescent-labeled probe (green) and Dapi staining (blue).

We found that *linc-MYH* is expressed in fast-type skeletal muscles (GP, TA and EDL), but not in SOL, brain, kidney, heart or fat tissues, an expression pattern which parallels that of the fast-fiber *Myh4* (Figure 4A). This suggested that *linc-MYH* is only expressed following robust nuclear accumulation of Six1, as takes place in the nuclei of *MYH4* myofibers [11], and that the weaker nuclear accumulation of Six1 observed in SOL myonuclei does not allow efficient Six1 binding on the *MYH* enhancer and *linc-MYH* expression. We used luciferase reporter transfection assays (as described above) to test the requirement for Six binding on the *MYH* enhancer to activate *linc-MYH* expression. These transient transfection assays, performed in adult TA, show that the *MYH* enhancer activates *linc-MYH* expression in a Six-dependent manner, as measured two weeks after electroporation (Figure 4B). *lincRNAs* can localize in cytoplasm [16] or as a single focus [19] or multiple foci [20] in nuclei. To analyze *linc-MYH* localization in skeletal muscle fiber, we performed fluorescent *in situ* hybridization (FISH), using *linc-MYH* sense and antisense RNA on isolated myofibers from fast EDL. Intranuclear localization of *linc-MYH* was observed with the antisense *linc-MYH* RNA probe, with approximately 10 *linc-MYH* foci per nucleus (n = 10, Figure 4C), while the sense *linc-MYH* RNA probe gave no signal (data not shown). We concluded from these experiments that *linc-Myh* RNA accumulates at specific sites in the nucleus of fast myofibers.

### *linc-MYH* coordinates fiber-type gene expression

**Figure 5:**
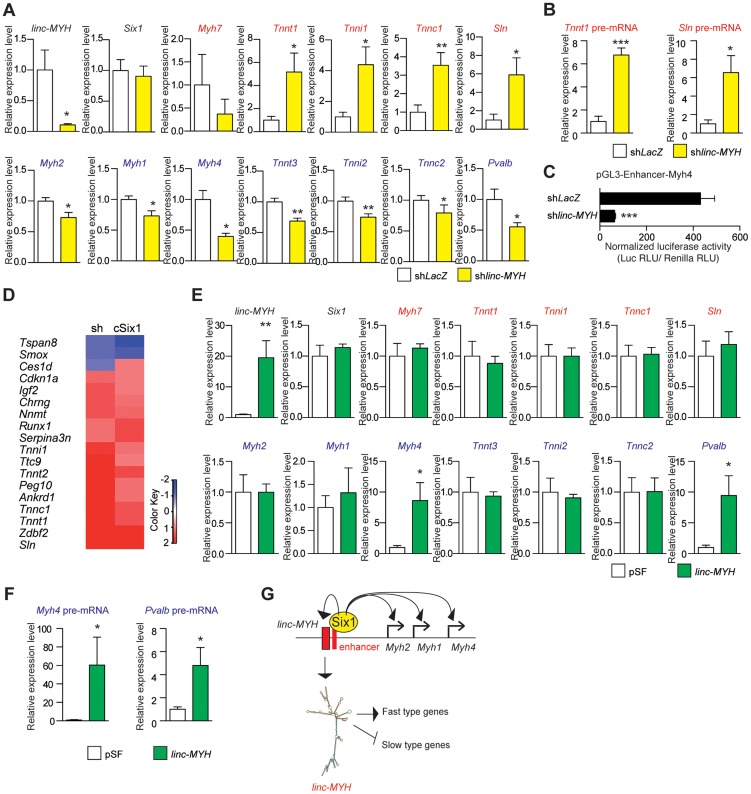
Slow-type gene expression is suppressed by *linc-MYH*. (A) qPCR experiments revealing mRNA expression levels of *linc-MYH*, *Six1*, slow-type genes (red) and fast-type genes (blue) in TA muscles expressing a shRNA directed against *linc-MYH* or *LacZ* (n = 5). (B) qPCR experiments revealing pre-mRNA expression of the *Tnnt1* and *Sln* slow genes in TA muscles expressing a shRNA directed against *linc-MYH* or *LacZ* (n = 5) (C) Luciferase activity of pGL3-Enhancer-*Myh4* in TA muscles expressing a shRNA directed against *linc-MYH* or *LacZ* (n = 4). (D) Microarray analysis of TA muscles transfected by shRNA against *linc-MYH*: a heat map of genes (red) upregulated to more than double the levels observed in c*Six1*KO. (E) qPCR experiments revealing mRNA expression levels of *linc-MYH*, *Six1*, slow-type genes (red) and fast-type genes (blue) in Soleus muscles expressing *linc-Myh* or the empty vector pSF (n = 4). (F) qPCR experiments revealing pre-mRNA expression of the *Myh4* and *Pvalb* fast genes in Soleus muscles expressing *linc-Myh* or the empty vector pSF (n = 4) (G) A model of *Six1* controlling the expression of the different *MYH* and of *linc-MYH* at the fast *Myh* locus in fast myofibers. Below, the hypothesis explaining the *linc-MYH* mode of action, as supported by the transcriptomic-wide analysis performed after *linc-MYH* knockdown in fast TA. *P<0.05, **P<0.01, ***P<0.001.

Due to the number of *linc-MYH* foci observed in fast type nuclei, we hypothesized that *linc-MYH* could act in trans [17] to control gene expression in fast myofibers. To test this theory, we used electroporation to introduce a shRNA against *linc-MYH* (sh*linc-MYH*) in TA muscle and analyzed the transfected samples after fourteen days. This method yielded the efficient knockdown of *linc-MYH*, with a 90% reduction of its expression (Figure 5A). To identify the consequences of *linc-MYH* knockdown and understand its mode of action, RNA samples from sh*linc-MYH* transfected adult TA were analyzed by Affymetrix microarrays (Figure 5D and Table S1), and validated by qPCR experiments (Figure 5A). The expression of *linc-MYH* was significantly lower in the absence of *Six1*, but *Six1* expression was not affected by the absence of *linc-MYH*. Knockdown of *linc-MYH* led to robust gene expression modification; this knockdown strongly upregulated the expression of numerous slow genes (such as *Sln*, *Tnni1*, *Tnnc1* and *Tnnt1*) and moderately downregulated the expression of several fast genes, (including *Myh4*, *Tnnt3*, *Tnni2* and *Pvalb*) (Figure 5A). We noted that the expression of *Sln* and other slow genes remained far lower in *linc-MYH* knockdown TA than in wild type Soleus. This showed that *linc-MYH* knock down in TA is not sufficient to achieve the full transcription efficiency of these slow genes as is observed in SOL, where calcineurin and several kinases are required to achieve their efficient transcription [27]. In addition, and contrary to what was observed previously in the muscles of *cSix1* KO mice, slow *Myh*7 expression level did not change in *linc-MYH* knock down TA. We next tested whether the transcription rate of *Sln* and *Tnnt1* slow genes was modified in *linc-MYH* knock down TA by measuring their pre-mRNA expression level. As can be seen in Figure 5B we observed an upregulation of pre-mRNA of these slow genes after *linc-MYH* knock down, which is proportional to their respective mRNA accumulation, demonstrating that their transcription is increased after *linc-MYH* knock down in TA, and showing that *linc-Myh* can act in trans to decrease their transcription. Since we also observed a moderate down-regulation of the expression of fast genes in *linc-MYH* knock down TA, among which *Myh4*, we next tested whether the activity of the pGL3-Enhancer-*Myh4* reporter could be modulated by the absence of *linc-MYH*. Two weeks after cotransfection of pGL3-Enhancer-*Myh4* and sh*linc-MYH* in TA, we observed a statistically significant decrease of Luciferase activity, demonstrating the requirement of *linc-MYH* for efficient transcription of *MYH4* (Figure 5C). Altogether these experiments show that accumulation of *linc-Myh* in the nuclei of fast myofibers participates in the transcriptional silencing of slow genes and is required for the full activation of fast genes.

### Transcriptomic analysis of *cSix1* and *linc-MYH*

We compared the networks of genes under the control of *linc-MYH* and of Six1 homeoprotein in adult muscles by the transcriptomic analysis of c*Six1* and *linc-MYH* knockdown (Figure 5D and Table S2). We found that the six genes whose expression was the most increased in the *linc-MYH* knockdown were also significantly upregulated in *cSix1 KO* muscles (Figure S5). Besides slow muscle genes, two genes, *Ankrd1* and *Peg10*, were more severely upregulated in the *linc-MYH* knockdown line (10 and 8 times, respectively) than in c*SIX1* mutant myofibers (by 2.8 and 1.5 times, respectively). These non slow-type genes could be exclusively repressed by *linc-MYH* in adult fast myofibers since there is a stronger downregulation of *linc-MYH* accumulation after its knockdown than in c*Six1* mutant myofibers. Transcriptomic analysis of adult myofibers deprived of either *Six1* or of l*inc-Myh* identified a strong qualitative and quantitative correlation in the expression of specific genes between *linc-MYH* knockdown and *cSix1* adult mutant myofibers. The expression of slow muscle genes was 3 to 10 fold higher in *linc-MYH* knockdown samples, and 5 to 25 fold higher in c*Six1*KO samples, than in the wild type. We further showed that *linc-MYH* lies downstream of *Six1* in the Six myogenic pathway and helps to repress slow muscle genes in fast myofibers. The downregulation of all fast-type genes (other than *Myh*4), and the upregulation of slow-type genes, was weaker in the *linc-MYH* knockdown than in the *Six1*cKO line. *Six1* may control several inhibitory pathways, including the *linc-MYH* pathway, to prevent slow-type genes expression in adult fast myofibers. During fetal development, at a stage where *linc-MYH* expression is not yet activated, *Six1/4* increases the nuclear accumulation of the slow muscle repressors Sox6 and HDAC4 to repress slow muscle gene expression [9],[28],[29]. In accordance with this, the expression of the slow genes *Myh7*, *Sln*, *Tnni1* and *Tnnt1* is upregulated in the muscle-specific *cSox6* mutant [30]. This demonstrates that *linc-MYH* and Sox6, lying both downstream of Six1, directly participate in the downregulation of *Sln*, *Tnni1* and *Tnnt1* in fast myofibers. However, the repression of slow *Myh7* in fast myofibers acts by a Six1-Sox6 dependent, but *linc-MYH* independent, repression mechanism. In this study, we observed that the levels of fast muscle gene expression decreased by 2–3 folds in the *linc-MYH* knockdown, with the highest decrease found for *Myh*4 expression. The expression of these genes decreased by a factor of 1.3 to 2.5 in c*Six1*KO, with the highest decrease found for *Pvalb* expression (Figures 3E and 5A). The presence of Six4 and Six5 proteins in adult myofibers [11], which have the same DNA binding specificity as Six1, could compensate its absence in c*Six1* KO animals and enable the activation of downstream fast muscle targets. In this case, *linc-MYH* expression could be preferentially dependent upon Six1, rather than on Six4 or Six5. Altogether, these experiments suggest that the accumulation of *linc-MYH* transcripts in the nuclei of fast myofibers facilitates the regulation of a network of genes that drive myofiber specialization via the same pathway as *Six1* and downstream of this transcription factor.

### Forced expression of *linc-MYH* in adult slow Soleus

To test the possibility that *linc-MYH* could modulate the expression of specific muscle genes, we forced its expression in myogenic C2 cells and in primary myoblasts, where endogenous *linc-MYH* expression was faintly detectable even in myotubes four days after their differentiation (data not shown). Transfection of a 13 kb genomic fragment encompassing the whole *linc-MYH* gene lead to efficient *linc-MYH* RNA accumulation in myotubes, but after this forced expression, we were unable to detect any modification in the expression of slow or fast type genes (data not shown). These results suggest that specific cofactors of *linc-MYH* required for its appropriate functioning are lacking in cultured myotubes in culture, in agreement with the expression of *linc-Myh* only in adult fast type fibers. To circumvent the limitations of cultured cells, we turned to *in vivo* experiments in the Soleus in which *linc-Myh* is weakly expressed. Two weeks after *linc-MYH* gene transfection in the Soleus we observed that *linc-MYH* RNA accumulates up to approximately 80% of its expression level in TA (Figure 5E). We observed a selective upregulation of *Myh4* and *Pvalb* mRNAs which were increased to approximately one-third their expression level observed in TA, while mRNA for slow genes remained unchanged (Figure 5E). To test whether the increase in *Myh4* and *Pvalb* mRNA was due to an increased transcription of their genes, and potentially exclude a mechanism implicating mRNA stabilization, we measured pre-mRNA accumulation. As can be seen in Figure 5F, the transcription of these two fast genes is upregulated in the Soleus samples expressing *linc-MYH* proportionally to their mRNA accumulation, demonstrating that *linc-Myh* can work in trans and allow efficient activation of the transcription of specific fast genes. Absence of down regulation of the expression of slow genes in Soleus myofibers expressing *linc-Myh* suggests that, like in cultured myotubes, *linc-Myh* RNA needs specific protein-binding partners to achieve its function. Such specific protein-binding partners may be absent in Soleus as well as in cultured myotubes. Nuclear long non coding RNA are known to guide chromatin modifiers to specific gene loci, and by recruiting histone modifiers or DNA methyltransferase to modulate their transcription rate [31]. Potential *linc-Myh* protein partners expressed differentially in fast and slow adult myofibers may explain how *linc-Myh* efficiently represses the transcription of slow genes and activates the transcription of fast genes in fast myofibers, while in slow myofibers its forced expression is only able to activate the transcription of fast genes.

## Discussion

The commitment and maintenance of muscle fiber fast sub type specialization relies on the specific expression of one of the fast Myosin heavy chain gene present at the fast *Myh* locus, and of specific isoforms of sarcomeric genes [1],[2],[4]. Myosin heavy chains are the primary determinant of the efficiency of muscle contraction. In this manuscript, we identified a novel mechanism for the specialization of the fast-myofiber subtype. We show that the long intergenic non-coding RNA *linc-MYH* and fast *MYH* genes, both of which are essential for myofiber specification, share a common enhancer which is regulated by Six1 homeoproteins. The *linc-MYH* specifically accumulates in nuclei of adult fast myofibers. Its function, as revealed here by *in vivo* knockdown and transcriptome-wide analysis, is to prevent slow-type muscle gene transcription and increase fast-type muscle gene expression in fast-type myofibers. We found *linc-MYH* downregulates the transcription of genes associated with slow muscle contractile properties like the slow genes *Tnn* and *Sln* (a known repressor of Serca1/Atp2a1 protein [32],[33] involved in Ca^++^ reuptake by the sarcoplasmic reticulum). These genes, which belong to the muscle contractile machinery and are repressed in adult fast myofiber, are positively controlled by Six1 in myogenic C2 cells [34], where *linc-MYH* expression is not detected. This suggests that their expression in adult fast myofiber may be restricted by an additional level of regulation involving the *Six1-linc-MYH* axis. As a result of our study we suggest that Six1 controls the acquisition of fast-type myofiber mechanical properties by binding to a single enhancer region of the fast *Myh* locus. It promotes the coordinated expression of fast *Myhs* and that of a strong repressor of genes controlling slow contractile properties. The modulation of Six activity (depending on fiber-type) facilitates changes in the expression levels of the fast genes *Myh* and *Tnn*; these changes are required for the formation of efficient sarcomeric units and the appropriate Ca^++^ cycling and excitation/contraction/relaxation coupling [1]–[4]. The *Myh* enhancer element therefore connects distinct regulatory hubs to achieve ultimate muscle fiber specialization. In this context, *linc-MYH* functions as an end-of-the-chain control element, conveying information on the state of fast *Myh* enhancer activity to repress slow-type specific genes and coordinates a finer level of regulation. This genomic organization at the fast *Myh* locus is reminiscent of the slow *Myh7* locus where two microRNA miR-208b and miR-499 involved in fast myofiber program repression are co-regulated with *Myh7* [35]. The precise molecular interactions between *linc-Myh* and higher order chromatin modifying complexes remains to be identified, to explain how *linc-Myh* coordinates the activation of target genes at specific sites in the nucleus, and the repression of others.

## Materials and Methods

### Mice, ethics statement

Animals were bred and handled as recommended by European Community guidelines. Experiments were performed in accordance with the guidelines of the French Veterinary Department. *cSix1*KO mice were obtained by breeding the *Six1*-*LoxP* mice [26] and transgenic mice expressing a CRE recombinase under the control of the human skeletal actin promoter (HSA) [25].

### ChIP experiments

GP and TA muscles of 2 months old mice were minced with scissors just after sampling and fixed in 1% formaldehyde for 10 minutes. Formaldehyde was quenched by addition of 0.125 M glycine, and muscles were washed twice in PBS. The muscles were incubated on ice in lysis buffer (10 mM Tris-HCl pH 7.9, 85 mM KCl, 0.5% NP40, protease inhibitors (cOmplete, Roche)) for 10 minutes and homogenized with a mortar and, subsequently with a Dounce homogenizer. The nuclei were obtained by centrifugation, incubated in SDS lysis buffer (50 mM Tris-HCl pH 8, 10 mM EDTA, 1% SDS, protease inhibitors) for 10 minutes, and sonicated in a Bioruptor apparatus (Diagenode). The debris were removed by centrifugation. The sonicated DNA was incubated with 1 µg of *Six1* antibodies (HPA001893, Sigma) under agitation at +4°C overnight. 20 µl of Dynabeads protein G (Invitrogen) were added to the samples and incubated under rotation at +4°C for 1 hour. The beads were washed with low-salt buffer (2 mM EDTA, 20 mM Tris-HCl pH 8, 150 mM NaCl, 1% TritonX-100, 0.1% SDS), high salt buffer (2 mM EDTA, 20 mM Tris-HCl pH 8, 0.5M NaCl, 1% TritonX-100, 0.1% SDS), LiCl buffer (1 mM EDTA, 10 mM Tris-HCl pH 8, 0.25M LiCl, 1% NP40, 1% deoxycholate) and TE buffer (1 mM EDTA, 10 mM Tris-HCl pH 8). The DNA was eluted with elution buffer (1% SDS, 0.1 M NaHCO_3_) containing 0.1 mg/ml proteinase K (Invitrogen) at 62°C for 2 hours, and, proteinase K was inactivated by incubation at 95°C for 10 minutes. The DNA was finally purified with MinElute PCR purification kit (Qiagen). The amount of specific amplified DNA is normalized by *beta-Actin* promoter amplification. The sequences of the oligonucleotides used in this study are as follows. Enh 1F, 5′-ATC TCC ACC TCC CTC CAA CT; Enh 1R, 5′-ACC CCC TAG CTT TGA CAG GT; Enh 2F, 5′-AAT CTG ACG ACA GGG TGA GC; Enh 2R, 5′-GGT CGC CTG ACC TGA TAG AG; AldoaF, 5′-CTC TCA AGG CAA ACC AAA GC; AldoaR, 5′-CCA GTG TCC CAG ACC TTC TC; ActbF, 5′-TGT TAC CAA CTG GGA CGA CA; ActbR, 5′-ACC TGG GTC ATC TTT TCA CG, NCF, 5′-ATC CTG CCC CAC TGT GTT AG; NCR, 5′-GCC AGC AAT TTG GTT TGA AT.

### 3C experiments

3C experiments were performed as described [36] with few modifications. Single myofibers were obtained from adult EDL muscles as previously described [26], cross-linked in 2% formaldehyde, 10 mM Tris-HCl pH 7.9, 85 mM KCl, 0.5% NP40 for 10 min at room temperature. Crosslinking reaction was quenched by 1 M glycine. Cross-linked myofibers were lysed for 10 minutes with lysis buffer (10 mM Tris-HCl pH 7.9, 85 mM KCl, 0.5% NP40) on ice, and the nuclei were harvested. Nuclei were resuspended in appropriate restriction enzyme buffer, 0.3% SDS and incubated for 1 hour at 37°C with shaking. Triton X-100 was added to 2%, and samples were incubated for 1 hour at 37°C. Samples were digested with Hind III overnight at 37°C. DNA ligation was performed for 4 hours at 16°C and for 30 minutes at room temperature. Cross-links were reversed, and DNA was then purified by phenol extraction and ethanol precipitation. To correct for the PCR amplification efficiency of different primer sets, a BAC clone containing the mouse *Myh* locus (RP23-61C14) was digested, ligated and used as control templates. Quantification of the data was performed by quantitative real-time PCR using the Lightcycler 480 probe master (Roche Diagnostic). The sequences of the oligonucleotides used in this study are given in Table S3.

### RNA preparation

TA, back, soleus and GP muscles were collected from *cSix1 KO* and control mice. Total RNAs were extracted by Trizol Reagent (Invitrogen) according to manufacturer's instruction.

### cDNA synthesis and qPCR

RNAs were treated with DNase I (Turbo DNA-free, Invitrogen) and were reverse transcribed with Superscript III kit (Invitrogen) according to manufacture's instruction. Reverse transcription was performed with 1 µg of total RNA. Quantitative real time PCR (Light Cycler 480, Roche) was performed using Light Cycler 480 SYBR Green I Master Kit (Roche) according to the manufacturer's protocols. PCR was performed for 40 cycles of 95°C for 15 seconds, 60°C for 15 seconds, and 72°C for 15 seconds. Genes expression level was normalized by the expression level of the housekeeping gene *Actb*. The sequences of the oligonucleotides used in this study are given in Table S4. Pre-mRNA qPCR experiments to measure RNA transcription rate were performed in the same conditions. Reverse oligonucleotides were complementary to intronic sequences, while forward oligonucleotides were complementary to exonic sequences. Samples without reverse transcription were used as controls, and signal due to contaminating DNA was subtracted to the values obtained with cDNA. We noticed that genomic DNA contamination was very low (less than hundred fold level of qPCR value observed with cDNA).

### Muscle contraction test

Skeletal muscle function was evaluated by measuring *in situ* muscle contraction, as described previously [37]. 12 week-old male mice were anesthetized (intraperitoneal injection of pentobarbital sodium, 50 mg/kg). Body temperature was maintained at 37°C using radiant heat. The distal tendon of the TA muscle was attached to an isometric transducer (Harvard Bioscience) using a silk ligature. The sciatic nerves were proximally crushed and distally stimulated by a bipolar silver electrode using supramaximal square wave pulses of 0.1 ms duration. Responses to tetanic stimulation (pulse frequency 50–143 Hz) were successively recorded. Maximal forces were determined at optimal length (length at which maximal force was obtained during the tetanus). Fatigue resistance was then determined after a 5-minutes rest period. The muscle was continuously stimulated at 50 Hz for 2 minutes (sub-maximal continuous tetanus), and the duration corresponding to a 20% decrease in force was recorded.

### RNA-FISH

Fluorescent-labeled antisense *linc-MYH* probes were synthesized according to manufacturer's instruction (FISH Tag RNA kit, Invitrogen). FISH experiments were performed on isolated EDL myofibres and images acquired on a Leica SP2 confocal microscope.

### Generation of shRNA against mouse *linc-MYH*

Five distinct shRNAs targeting mouse *linc-MYH* were designed, called sh*lincMYH*, and inserted into the psiSTRIKE hMGFP system (Promega). The efficiency of each shRNA was established by determination of *linc-MYH* transcript levels in TA muscles transfected by each sh*lincMYH*. The shRNA against 5′-TTC TGC TCA CCA CCT ACA ATT-3′ sequence was selected for the knockdown experiment. For knock down experiments using sh*lincMYH*, a plasmid coding for sh*LacZ* was electroporated in the contra-lateral TA as a negative control.

### Electroporation

*In vivo* transfections were also carried out on 10-weeks old C57Bl6 mice. For each experimental conditions, three to five *Tibialis anterior* (TA) or *Soleus* (Sol) muscles belonging to different mice were used. Under isoflurane anesthesia, legs were shaved and muscles were pre-treated by injection of a sterile 0.9% NaCl solution containing 0.4 U of bovine hyaluronidase/µl two hours before plasmid injection. Ten µg of shRNA-expressing vector were introduced into TA muscles of 8 week-old mice by electroporation as previously described [11]. Two weeks following electroporation, TA myofibers expressing GFP were dissected under a Nikon SMZ1500 stereo microscope and frozen in liquid nitrogen before processing for Luciferase assays or RNA purification.

### Immunohistochemistry

TA, soleus and gastrocnemius muscles were embedded in cryomatrix and quickly frozen in isopentane cooled with liquid nitrogen. Cryostat sections (10 µm) were fixed in 4% PFA, washed in PBS, permeabilized with 0.1% Triton X-100 and left for 1 hour in blocking solution (1× PBS, 1.5% goat serum, 0.1% Triton X-100). Rabbit poly-clonal antibodies directed against Laminin (Z0097, Dako) (1/100 dilution), and monoclonal antibodies against *MYH*7 (NOQ7.5.4D, Sigma) (1/1000 dilution), *MYH*2 (SC-71, Developmental Studies Hybridoma Bank) (1/20 dilution) and against *MYH*4 (BF-F3, Developmental Studies Hybridoma Bank) (1/20 dilution) were applied overnight at 4°C to the treated sections. The next day, after three washes with 1× PBS containing 0.05% Tween-20, cryosections were incubated for 1 h with appropriate fluorescent secondary antibodies (Alexa Fluor 488 goat anti-rabbit IgG 1/1000 dilution, Alexa Fluor 594 goat anti-mouse IgG 1/1000 dilution, Invitrogen). After three washes with 1× PBS containing 0.05% Tween 20, samples were mounted in Vectashield mounting medium.

### Microarray

After validation of RNA quality with the Bioanalyzer 2100 (using Agilent RNA6000 nano chip kit), 50 ng of total RNA were reverse transcribed following the Ovation PicoSL WTA System (Nugen). Briefly, the resulting double-strand cDNA was used for amplification based on SPIA technology. After purification according to Nugen protocol, 5 µg of single strand DNA was used for generation of Sens Target DNA using Ovation Exon Module kit (Nugen). 2.5 µg of Sens Target DNA were fragmented and labelled with biotin using Encore Biotin Module kit (Nugen). After control of fragmentation using Bioanalyzer 2100, the cDNA was then hybridized to GeneChip Mouse Gene 1.0 ST (Affymetrix) at 45°C for 17 hours. After overnight hybridization, the ChIPs were washed using the fluidic station FS450 following specific protocols (Affymetrix) and scanned using the GCS3000 7G. The scanned images were then analyzed with Expression Console software (Affymetrix) to obtain raw data (cel files) and metrics for Quality Controls. The analysis of some of these metrics and the study of the distribution of raw data show no outlier experiment. RMA normalization was performed using R and normalized data was subjected to statistical tests.

### EMSA

EMSA was carried out with *Six1* full-length mouse cDNA cloned into the pCR3 vector (Clontech) as previously described [38]. Recombinant mouse *Six1* protein was obtained with a T7 transcription/translation kit (Promega). The oligonucleotide containing double-stranded myogenin MEF3 site was incubated with recombinant proteins. Competition experiments were performed in the presence of a ten-fold and hundred-fold molar excess of unlabeled identified *Myh* enhancer MEF3 sites (Enh1 to Enh6) or mutated *Myh* MEF3 sites (mtEnh1 to mtEnh6), or *Myogenin* promoter NFI or MEF3 sites. The sequences of the oligonucleotides used are as follows, the MEF3 consensus sequence is underlined; Enh1F 5′-CTC TTG GGT AAC TGG AGC CCC TC-3′. Enh1R 5′-GAG GGG CTC CAG TTA CCC AAG AG-3′. Enh2R 5′-GGT TGA CTT AGA TTT CCT TAT GA-3′. Enh2F 5′-TCA TAA GGA AAT CTA AGT CAA CC-3′. Enh3F 5′-TGT AAG AGA AAC TGA AAT AAA AT-3′. Enh3R 5′-ATT TTA TTT CAG TTT CTC TTA CA-3′. Enh4F 5′-GGG GTA AGA AAT CTG ACG ACA GG-3′. Enh4R 5′-CCT GTC GTC AGA TTT CTT ACC CC-3′. Enh5F 5′-CTA TCA GGT CAG GCG ACC TCA GT-3′. Enh5R 5′-ACT GAG GTC GCC TGA CCT GAT AG-3′. Enh6F 5′-CGT CAA GGA AAC CTT ATT CCA TC-3′. Enh6R 5′-GAT GGA ATA AGG TTT CCT TGA CG-3′. MyogF 5′-TGG GGG GGC TCA GGT TTC TGT GGC GT-3′. MyogR 5′-ACG CCA CAG AAA CCT GAG CCC CCC CA-3′. NF1F 5′-TAT CTC TGG GTT CAT GCC AGC AGG G-3′. NF1R 5′-CCC TGC TGG CAT GAA CCC AGA GAT A-3′. mtEnh1F 5′-CTC TTG GGT AGG ATC CGC CCC TC-3′. mtEnh1R 5′-GAG GGG CGG ATC CTA CCC AAG AG-3′. mtEnh2F 5′-GGT TGA CGA ATT CTT GCT TAT GA-3′. mtEnh2R 5′-TCA TAA GCA AGA ATT CGT CAA CC-3′. mtEnh3F 5′-TGT AAG ACC AAC TGA AAT AAA AT-3′. mtEnh3R 5′-ATT TTA TTT CAG TTG GTC TTA CA-3′. mtEnh4F 5′-GGG GTA AGA AGG ATC CCG ACA GG-3′. mtEnh4R 5′-CCT GTC GGG ATC CTT CTT ACC CC-3′. mtEnh5F 5′-CTA TCA GGT CGG ATC CCC TCA GT-3′. mtEnh5R 5′-ACT GAG GGG ATC CGA CCT GAT AG-3′. mtEnh6F 5′-CGT CAA GGA AGG ATC CTT CCA TC-3′. mtEnh6R 5′-GAT GGA AGG ATC CTT CCT TGA CG-3′.

### Western blot

Western blots were performed with protein extracts of GP and soleus muscles from c*Six1*KO mice and control mice as previously described [9]. 1∶1000 dilutions of anti-Six1 antibodies (HPA001893, Sigma) or anti-β−tubulin antibodies (2128, Cell Signaling) were used.

### Statistical analysis

All graphs represent mean values ± SEM. Significant differences between mean values were evaluated using two-tailed, unpaired Student's t test (when two groups were analyzed) or one-way ANOVA followed by Student Newman-Keuls test (for three or more groups).

### Plasmids construction

For the construction of the pGL3-*Myh2/1/4*, c57bl6N mouse DNA was first used as a template to clone 1.1 kbp promoters of *Myh2/1/4* with forward KpnI/SacI 5′-TTC AGA AAC TGC ATC ACT TAA A-3′ and reverse MluI, 5′-GCA GCT CGG GCA GTG GCC AGT GT-3′, forward KpnI/SacI 5′- CAT ATC TGC ATC TCT AGA TAC C-3′ and reverse MluI, 5′- GGC AGC AGC AGC CAG GAT GTG T-3′, forward KpnI/SacI 5′- ACC GCT AGC CTT GAG CCT TTG-3′ and reverse MluI, 5′- ATA GCG AGA GCC CTT TGT TCT C-3′, respectively. *Myh2/1/4* promoter fragments were subsequently inserted into an KpnI-MluI digested pGL3 basic plasmid. For the construction of the pGL3-Enhancer-*Myh2/1/4*, mouse DNA was first used as a template to clone the enhancer with forward KpnI 5′- GCG TTT CTA ATT CGG CTT GAA C-3′ and reverse SacI, 5′- CAT TTC CTT CCT CTA AAG GCT CTT TATT C-3′. This enhancer fragment was subsequently inserted into KpnI-SacI digested pGL3-*Myh2/1/4* plasmids. For the construction of the pGL3-mutEnhancer-*Myh2/1/4*, the six MEF3 sites of the enhancer were mutated as follows; MEF3 1: 5′-GTAACTGGA to 5′-GTAGGATCC; MEF3 2: 5′-TTAGATTTC to 5′-GAATTCTTG; MEF3 3: 5′-GAAACTGAA to 5′-CCAACTGAA; MEF3 4: 5′-GAAATCTGA to 5′-GAAGGATCC; MEF3 5: 5′-GTCAGGCGA to 5′-GTCGGATCC; MEF3 6: 5′-GAAACCTTA to 5′-GAAGGATCC. All plasmids sequence was confirmed by sequencing.

For the construction of the pSF-pA-CMVe-*linc-MYH*, genomic DNA fragment containing *linc-MYH* was obtained from digestion of a BAC clone containing (RP23-61C14) by BsaWI and AvrII. The 13.3 kbp DNA fragment was subsequently inserted into a SpeI-XmaI digested pSF-pA-CMVe plasmid. For *linc-Myh* gain of function experiments, the empty pSF-pA-CMVe plasmid was electroporated in the contra-lateral Soleus as a negative control. For the construction of the pGL3-*Actb*, pGL3-*Sln and* pGL3-*Tnni1*, mouse DNA was first used as a template to clone the promoters of *Actb*, *Sln*, and *Tnni1* with forward 5′- TTT CTC TAT CGA TAG GTA CCT TTG AGC TCC TGA CCC CGT GTG TAG CTC T-3′ and reverse 5′- GAG CCC GGG CTA GCA CGC GTA AGG AGC TGC AAA GAA GCT G-3′, forward 5′- TTT CTC TAT CGA TAG GTA CCT TTG AGC TCT ACC GAC TAT CAT GCC CAC A-3′ and reverse MluI, 5′- GAG CCC GGG CTA GCA CGC GTC AGG CTA CCA AGG ACC TCA G-3′, forward 5′- TTT CTC TAT CGA TAG GTA CCT TTG AGC TCC TGG GAT TTG AAC CCA TGA C-3′ and reverse 5′- GAG CCC GGG CTA GCA CGC GTC CTC ACC ACA GAC TGC AGA G-3′, respectively. *Actb*, *Sln*, and *Tnni1* promoter fragments were subsequently inserted into a KpnI-MluI site of pGL3 basic plasmid by GeneArt kit (Life Technologies). For the construction of the pGL3-Enhancer-*Actb*, pGL3-Enhancer-*Sln and* pGL3-Enhancer-*Tnni1*, the enhancer fragment was subsequently inserted into an KpnI-SacI site of pGL3-*Actb*, pGL3-*Sln and* pGL3-*Tnni1*plasmids. All plasmids sequence were confirmed by sequencing.

### Luciferase assays

Two µg of Luciferase-expressing vector and one hundred ng of pRL-TK vector (Promega) were introduced into TA muscles of 8 week-old mice by electroporation as previously described [11]. Two weeks following electroporation, the TA muscles were dissected and frozen in liquid nitrogen before processing. The TA muscles were homogenized in Passive Lysis Buffer (Dual-Luciferase Reporter Assay System, Promega) and rotated for 15 minutes. The homogenate were centrifuged to remove debris, and the supernatant was used for measurement of Luciferase activity according to manufacture's instruction (Dual-Luciferase Reporter Assay System, Promega).

### Computational analysis

In order to computationally identify MEF3 binding sites, we built a PWM (position-specific weight matrix) for MEF3, starting from a list of 15 binding sites (see Table S5) that were previously tested by Electrophoretic Mobility Shift Assay on the basis of their proximity to the MEF3 *Myogenin* consensus GAAACCTGA [10]. The PWM (shown in Figure S6 and Table S6) was generated by running the *de novo* motif finder Imogene [39] on small DNA fragments (see Table S5) that contained these binding sites. Imogene used phylogeny to enrich mouse set of DNA fragments with orthologs in 11 other mammalian sequenced genomes and to produce a refined PWM. The information content of the PWM (the genmot Sg parameter of Imogene) was set to 8.7 bits. Binding sites were predicted using a prediction threshold (the scangen Ss parameter of Imogene) of 9 bits and requiring conservation, as explained in [39].

## Supporting Information

Figure S1 Sequences of P1 and P2 boxes of the *Myh* enhancer. Sequences of P1 and P2 boxes of the *Myh* enhancer in mouse, rat, human, bovine and equinides species, and showing the sequence conservation of the six MEF3 sites and E boxes. Coordinates are in mm9 assembly.
(TIF)

Figure S2 Predicted linc-MYH structure. Predicted minimum free energy (MFE) structure of the 1050 nt long linc-MYH, as determined by RNAfold [40]. The color encodes base-pair probabilities.
(TIF)

Figure S3 (A). Competitive Electromobility shift assays. Competitive Electromobility shift assays performed with recombinant Six1 proteins and labeled Myogenin MEF3 oligonucleotide and 10 or 100 fold molar excess of unlabelled oligonucleotides containing Myogenin MEF3 or NF1 site, with MYH MEF3 sites (1, 2, 3, 4, 5, 6) or with mutated MYH MEF3 sites whose sequence is presented on Figure S1 and in the Materials and Methods section. (B). qPCR experiments measuring the relative levels of *Sln* mRNA in adult wt TA and Sol. (C). Luciferase assays of adult TA transfected with *Sln*, *Tnni1* and *Actb* promoters with or without the *Myh* enhancer. TA sampling was performed two weeks after the electroporation, (n = 4). (D). Primary embryonic fibroblasts were transfected with Luciferase plasmids under the control of Myh4 promoter or Myh4 promoter linked with the Myh enhancer. After transfection, fibroblasts were cultured two days before sampling, (n = 3). *P<0.05, ***P<0.001.
(TIF)

Figure S4 Immunostaining of MYH proteins. Immunostaining of MYH7 (red), MYH2 (red), MYH4 (red) and laminin (green) in TA of 12 weeks old control and *cSix1KO* male mice.
(TIF)

Figure S5 Comparison between *cSix1* mice and sh*linc-MYH* treated mice. (*A*) Venn diagram showing the overlap between genes that are up-regulated more than five fold in sh*linc-MYH* and two fold in *cSix1* muscles. (p = 10^−17^ as given by a hypergeometric test). (*B*) Scatter plot of mRNA expression fold change (log2) as determined by microarray analysis of sh*linc-MYH* and *cSix1*. Genes that are up-regulated more than five fold in sh*linc-MYH* and two fold in *cSix1* muscles are indicated in red. (***C***) mRNA expression levels in sh*linc-MYH* knock-down and *cSix1* TA muscles, as measured by qPCR experiments. Ctrl (means ± SEM; n = 4), *cSix1* (means ± SEM; n = 4), sh*LacZ* (means ± SEM; n = 5), sh*linc-MYH* (means ± SEM; n = 5). (*D*) mRNA expression level of *Ankrd1*, *Zdbf2* and *Peg10* in back muscles of *cSix1* KO mice at E18.5, P2W, P4W and P8W, as determined by qPCR experiments, (means ± SEM; n = 3 to 6 for each point). *P<0.05, **P<0.01, ***P<0.001.
(TIF)

Figure S6 MEF3 PWM used in this study.
(TIF)

Table S1 Microarrays analysis of TA muscles electroporated by shRNA.
(XLS)

Table S2 Microarrays analysis of GP muscles of cSix1.
(XLS)

Table S3 Sequence of the oligonucleotides used for 3C.
(DOCX)

Table S4 Sequence of the oligonucleotides used for qPCR.
(DOCX)

Table S5 Coordinates (mm9) of the fragments used to learn the MEF3 PWM.
(DOCX)

Table S6 MEF3 frequency matrix.
(DOCX)
