# Correction: Six Homeoproteins and a linc-RNA at the Fast MYH Locus Lock Fast Myofiber Terminal Phenotype

**DOI:** 10.1371/journal.pgen.1004538

**Published:** 2014-07-01

**Authors:** The PLOS Genetics Staff

## Notice of Republication

In the HTML version of the manuscript there was an error in the title where the L of “linc-RNA” was replaced with an I. The first and last name of the corresponding author was also reversed. The pdf of the manuscript was unaffected. These errors are now corrected.
