# Supplementary figures and images for: Six Homeoproteins and a linc-RNA at the Fast MYH Locus Lock Fast Myofiber Terminal Phenotype
Source: PLoS Genet. 2014 May 22;10(5):e1004386. doi: 10.1371/journal.pgen.1004386 (PMC4031048; doi:10.1371/journal.pgen.1004386)

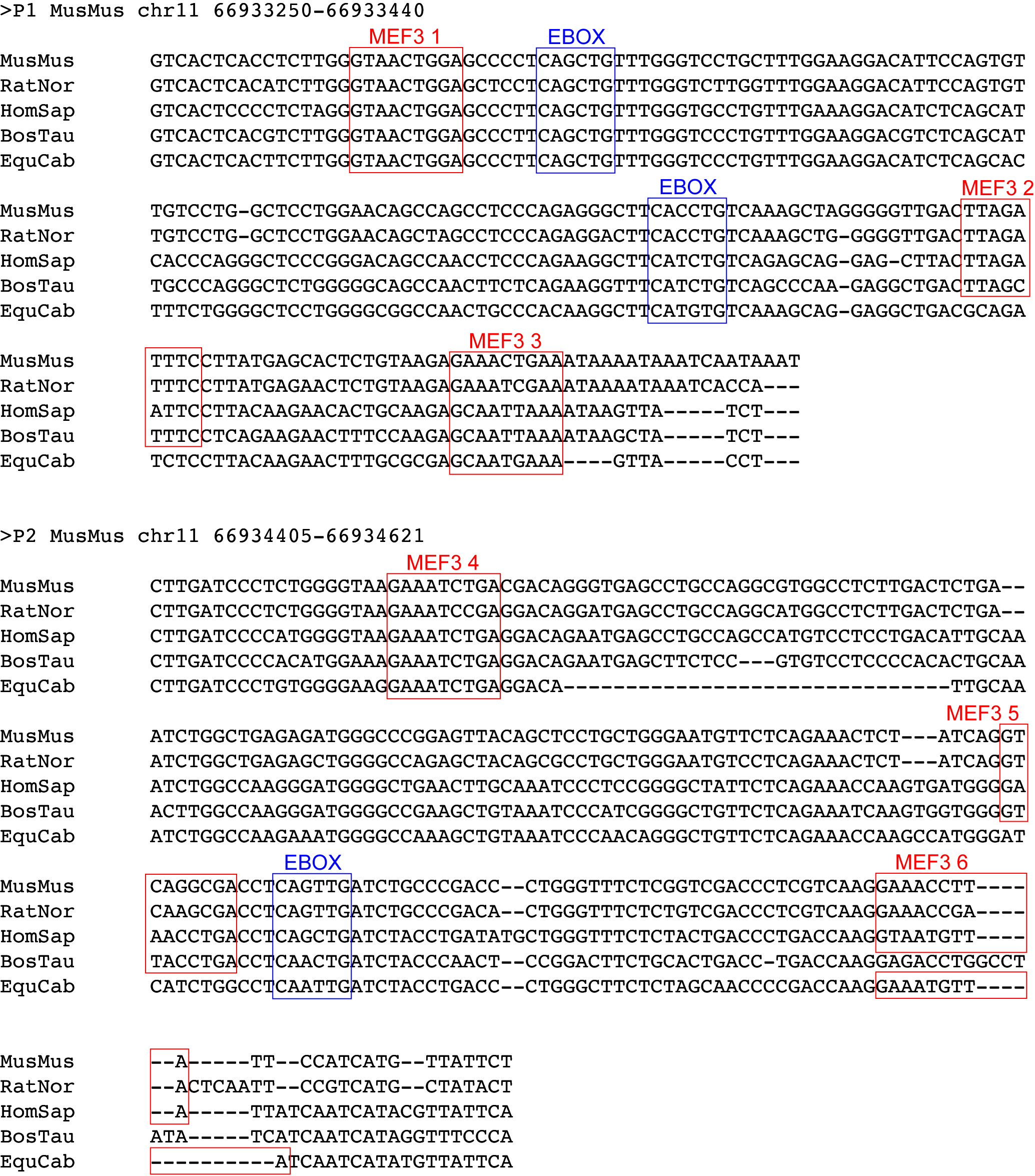

Supplement: Figure S1 — Sequences of P1 and P2 boxes of the Myh enhancer. Sequences of P1 and P2 boxes of the Myh enhancer in mouse, rat, human, bovine and equinides species, and showing the sequence conservation of the six MEF3 sites and E boxes. Coordinates are in mm9 assembly. (TIF) [file pgen.1004386.s001.tif]

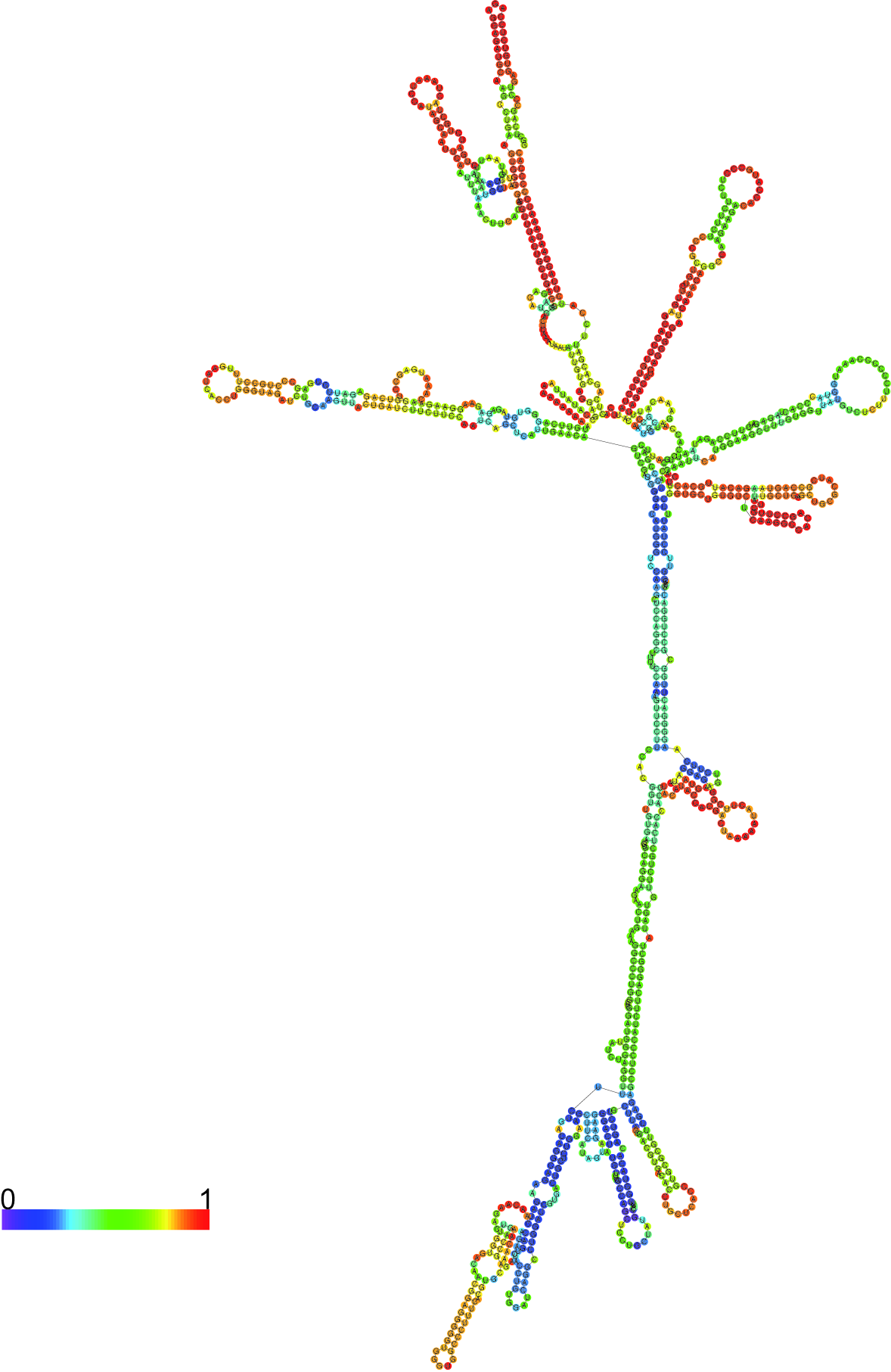

Supplement: Figure S2 — Predicted linc-MYH structure. Predicted minimum free energy (MFE) structure of the 1050 nt long linc-MYH, as determined by RNAfold [40]. The color encodes base-pair probabilities. (TIF) [file pgen.1004386.s002.tif]

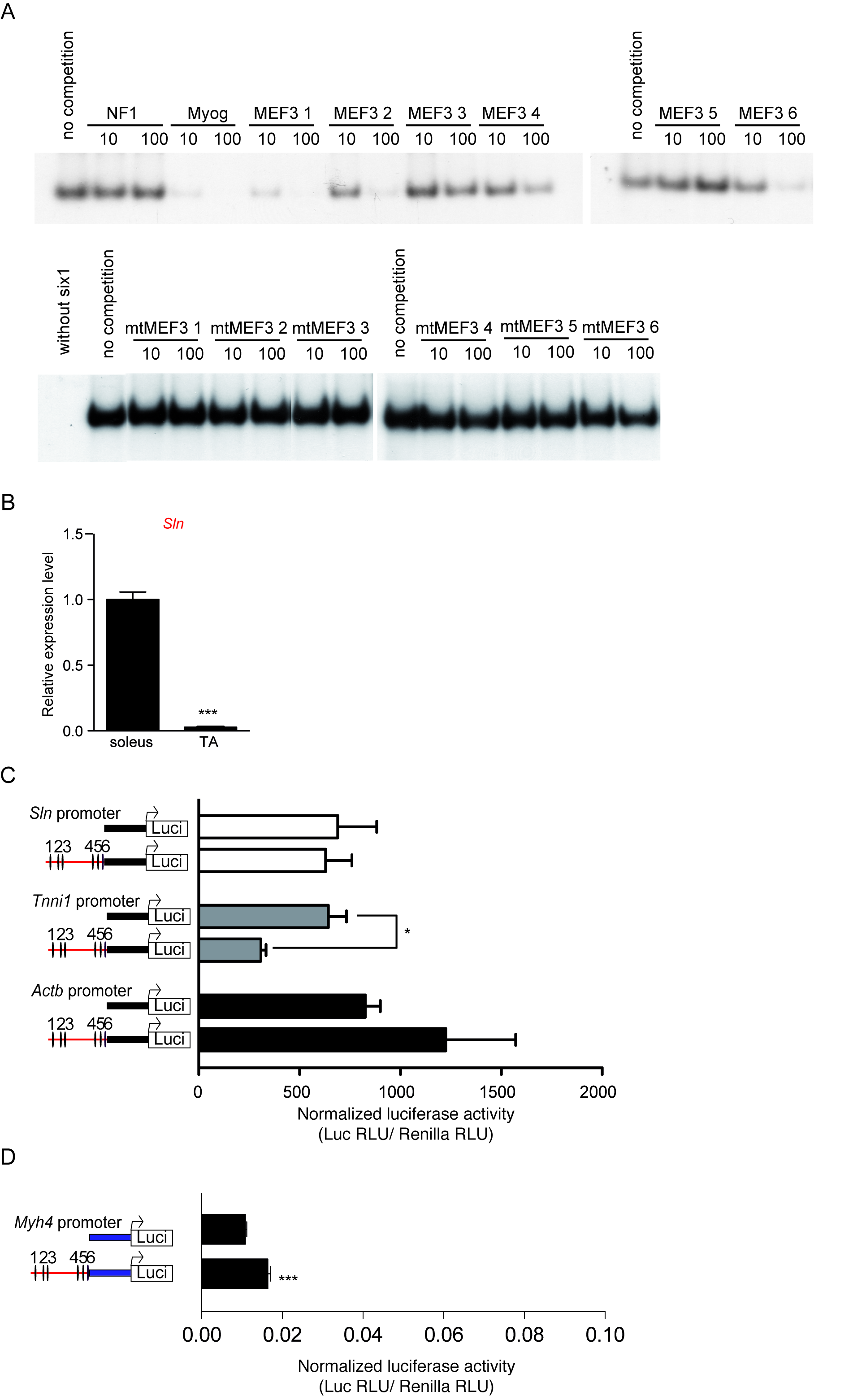

Supplement: Figure S3 — (A). Competitive Electromobility shift assays. Competitive Electromobility shift assays performed with recombinant Six1 proteins and labeled Myogenin MEF3 oligonucleotide and 10 or 100 fold molar excess of unlabelled oligonucleotides containing Myogenin MEF3 or NF1 site, with MYH MEF3 sites (1, 2, 3, 4, 5, 6) or with mutated MYH MEF3 sites whose sequence is presented on Figure S1 and in the Materials and Methods section. (B). qPCR experiments measuring the relative levels of Sln mRNA in adult wt TA and Sol. (C). Luciferase assays of adult TA transfected with Sln, Tnni1 and Actb promoters with or without the Myh enhancer. TA sampling was performed two weeks after the electroporation, (n = 4). (D). Primary embryonic fibroblasts were transfected with Luciferase plasmids under the control of Myh4 promoter or Myh4 promoter linked with the Myh enhancer. After transfection, fibroblasts were cultured two days before sampling, (n = 3). *P<0.05, ***P<0.001. (TIF) [file pgen.1004386.s003.tif]

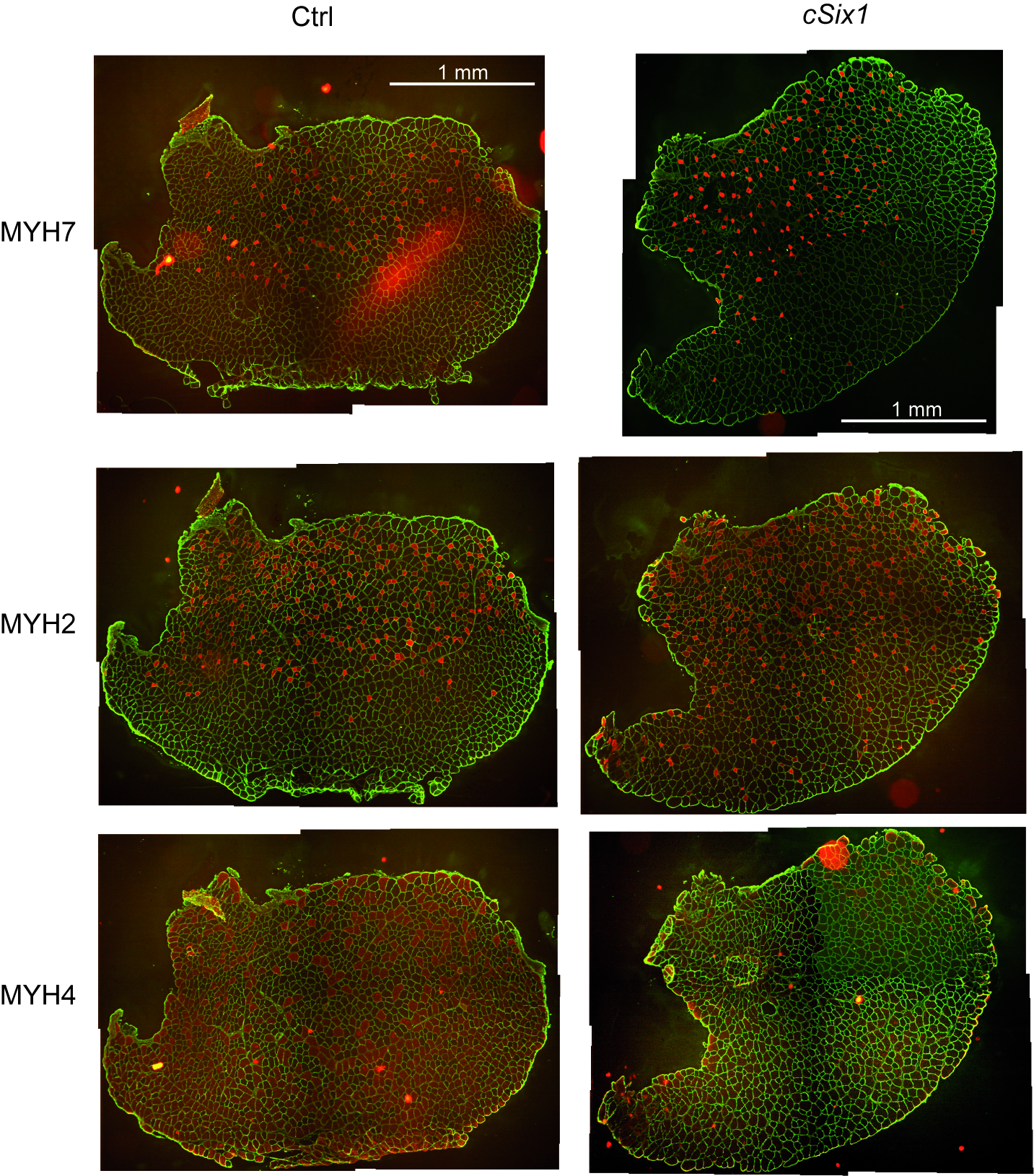

Supplement: Figure S4 — Immunostaining of MYH proteins. Immunostaining of MYH7 (red), MYH2 (red), MYH4 (red) and laminin (green) in TA of 12 weeks old control and cSix1KO male mice. (TIF) [file pgen.1004386.s004.tif]

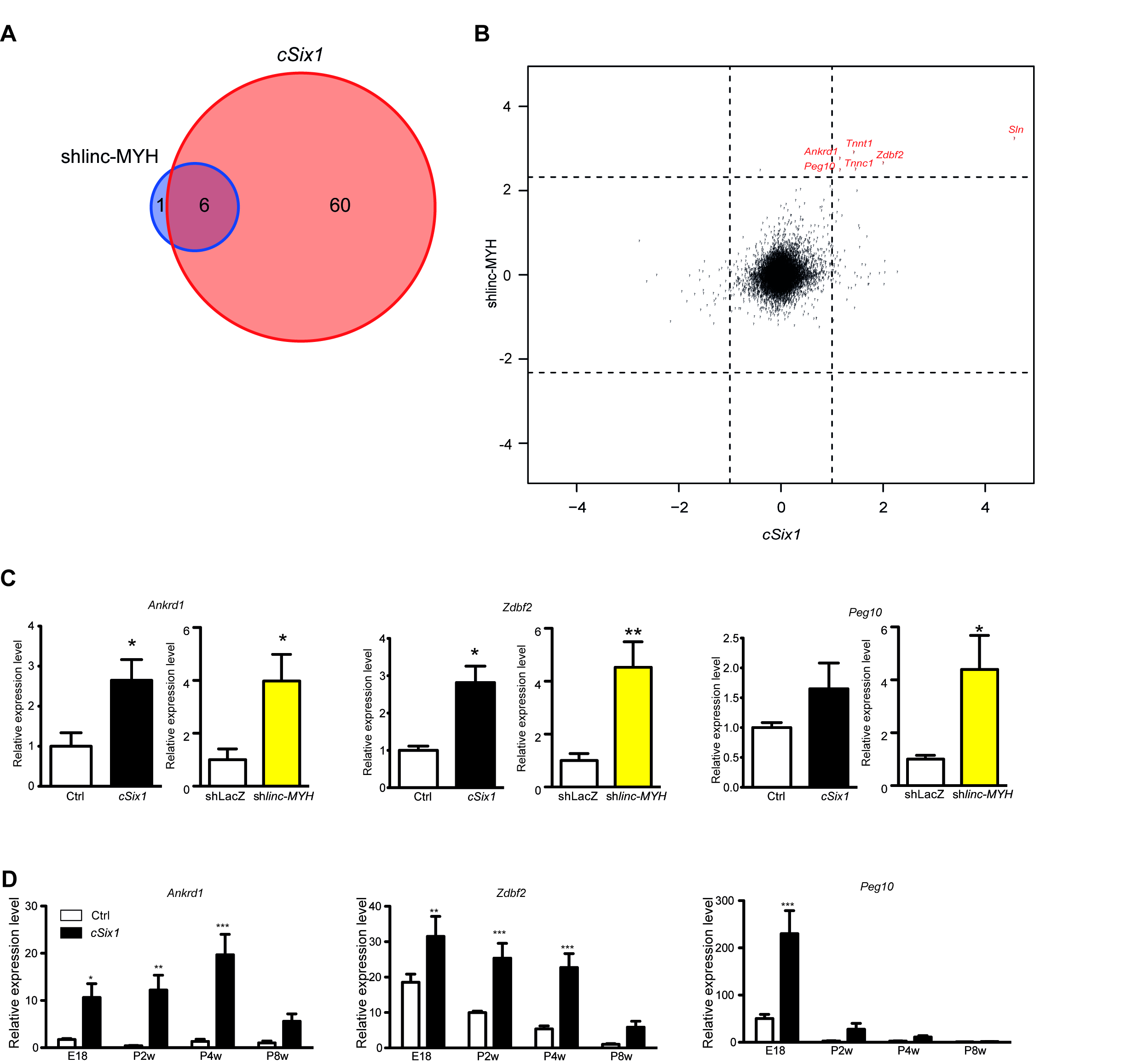

Supplement: Figure S5 — Comparison between cSix1 mice and shlinc-MYH treated mice. (A) Venn diagram showing the overlap between genes that are up-regulated more than five fold in shlinc-MYH and two fold in cSix1 muscles. (p = 10−17 as given by a hypergeometric test). (B) Scatter plot of mRNA expression fold change (log2) as determined by microarray analysis of shlinc-MYH and cSix1. Genes that are up-regulated more than five fold in shlinc-MYH and two fold in cSix1 muscles are indicated in red. (C) mRNA expression levels in shlinc-MYH knock-down and cSix1 TA muscles, as measured by qPCR experiments. Ctrl (means ± SEM; n = 4), cSix1 (means ± SEM; n = 4), shLacZ (means ± SEM; n = 5), shlinc-MYH (means ± SEM; n = 5). (D) mRNA expression level of Ankrd1, Zdbf2 and Peg10 in back muscles of cSix1 KO mice at E18.5, P2W, P4W and P8W, as determined by qPCR experiments, (means ± SEM; n = 3 to 6 for each point). *P<0.05, **P<0.01, ***P<0.001. (TIF) [file pgen.1004386.s005.tif]

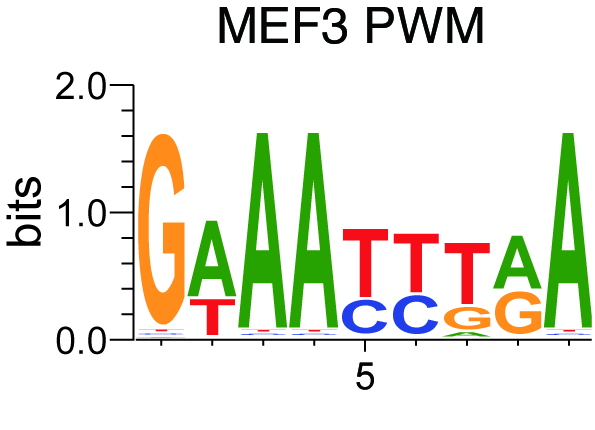

Supplement: Figure S6 — MEF3 PWM used in this study. (TIF) [file pgen.1004386.s006.tif]
