# Supplementary material for: Six Homeoproteins and a linc-RNA at the Fast MYH Locus Lock Fast Myofiber Terminal Phenotype
Source: PLoS Genet. 2014 May 22;10(5):e1004386. doi: 10.1371/journal.pgen.1004386 (PMC4031048; doi:10.1371/journal.pgen.1004386)
Supplement: Table S3 — Sequence of the oligonucleotides used for 3C. (DOCX) [file pgen.1004386.s009.docx]

**Table S3.** Sequence of the oligonucleotides used for 3C.

| name of oligonucleotides | Sequence (5'- 3') |
| --- | --- |
| probe | FAM- TCAGCTGCCCAGGGTGACCA- Tamra |
| Enh_3CF | CCAGCCTGTTCTGGGTACAT |
| MYH1_3CR | ACCCCTTGGAATGAGAGTGA |
| MYH2_3CR | TGAAGCAGTGTGGAACAAGC |
| MYH4_3CR | CCAAATTGGTTGATGCTCATT |
| 78038_3CR | CCTGACGCACCATGTCTAAA |
| 104838_3CR | CAGGATTTGGTAGGGGATGA |
| 106285_3CR | CGCACAGCCTAATGAAGACA |
| 134682_3CR | CCCTCTCATATGGTGCCAGT |
| 148019_3CR | GGTCACTGGAGGGATCTGAA |
