# Supplementary material for: Six Homeoproteins and a linc-RNA at the Fast MYH Locus Lock Fast Myofiber Terminal Phenotype
Source: PLoS Genet. 2014 May 22;10(5):e1004386. doi: 10.1371/journal.pgen.1004386 (PMC4031048; doi:10.1371/journal.pgen.1004386)
Supplement: Table S4 — Sequence of the oligonucleotides used for qPCR. (DOCX) [file pgen.1004386.s010.docx]

**Table S4.** Sequence of the oligonucleotides for qPCR.

| gene name | Forward (5'- 3') | Reverse (5'- 3') |
| --- | --- | --- |
| *Six1* | CTTTAAGGAGAAGTCTCGGG | TTCCAGAGGAGAGAGTTGAT |
| *MYH7* | AGGGCGACCTCAACGAGAT | CAGCAGACTCTGGAGGCTCTT |
| *MYH2* | CCAAGAAAGGTGCCAAGAAG | CGGGAGTCTTGGTTTCATTG |
| *MYH1* | CGGTGGTGGAAAGAAAGG | CAGGAGTCTTGGTTTCATT |
| *MYH4* | GCTTGAAAACGAGGTGGAAA | CCTCCTCAGCCTGTCTCTTG |
| *MYH3* | GCAAAGACCCGTGACTTCACCTCTAG | GCATGTGGAAAAGTGATACGTGG |
| *Tnnt1* | CCCCCGAAGATTCCAGAAGG | TGCGGTCTTTTAGTGCAATGAG |
| *Tnnt3* | GGAACGCCAGAACAGATTGG | TGGAGGACAGAGCCTTTTTCTT |
| *Tnni1* | ATGCCGGAAGTTGAGAGGAAA | TCCGAGAGGTAACGCACCTT |
| *Tnni2* | AGAGTGTGATGCTCCAGATAGC | AGCAACGTCGATCTTCGCA |
| *Tnnc1* | GCGGTAGAACAGTTGACAGAG | CCAGCTCCTTGGTGCTGAT |
| *Tnnc2* | ATGGCAGCGGTACTATCGACT | CCTTCGCATCCTCTTTCATCTG |
| *Sln* | GGTCCTTGGTAGCCTGAGTG | CGGTGATGAGGACAACTGTG |
| *Pvalb* | ATCAAGAAGGCGATAGGAGCC | GGCCAGAAGCGTCTTTGTT |
| *linc-MYH* | GTGCAGCCAGAACAAGACAG | CAAGATGGGAGGCTCTCAAA |
| *Aldoa* | actctctgctgaccgggctct | aatgcttccggtggactcat |
| *Zdbf2* | TAGCGGCTCTTTCGAGAGAC | CCCTGATCTGGGGAGTCAA |
| *Peg10* | TGCACAACTACACTGCCTTTATG | CTGGGCAATCATCTGGAATGC |
| *Ankrd1* | TGCGATGAGTATAAACGGACG | GTGGATTCAAGCATATCTCGGAA |
| *Actb* | GGCTGTATTCCCCTCCATCG | CCAGTTGGTAACAATGCCATGT |
| *Sln pre-mRNA* | AGACAGCCGGGGAGACAGCT | GCTAAGTACGAAGTCTTGCT |
| *Tnnt1 pre-mRNA* | CTTTGATTCCCCCGAAGATT | GGGTTTTCATGAAGGAAGCA |
| *Myh4 pre-mNRA* | TTTGATGCCAAGTCATCGGT | CTCCCACGTCTTGCTTTTAC |
| *Pvalb pre-mRNA* | TTCCCAACAGGATCTCCCAC | TCCAACCTCAGAGGCTACC |
