# Supplementary material for: Six Homeoproteins and a linc-RNA at the Fast MYH Locus Lock Fast Myofiber Terminal Phenotype
Source: PLoS Genet. 2014 May 22;10(5):e1004386. doi: 10.1371/journal.pgen.1004386 (PMC4031048; doi:10.1371/journal.pgen.1004386)
Supplement: Table S5 — Coordinates (mm9) of the fragments used to learn the MEF3 PWM. (DOCX) [file pgen.1004386.s011.docx]

**Table S5.** Coordinates (mm9) of the fragments used to learn the MEF3 PWM.

| Binding site (mouse) | Chromosome | Start | Stop |
| --- | --- | --- | --- |
| GAAACCTGA | 1 | 136186489 | 136186512 |
| GAAATCTAA | 1 | 78199417 | 78199441 |
| GTAACTGGA | 10 | 106980283 | 106980330 |
| GAAACCGGA | 7 | 53626660 | 53626684 |
| GTAATTTAA | 1 | 66968862 | 66968888 |
| GTAACTGGA | 7 | 133606880 | 133606904 |
| GTAACCTGA | 7 | 133605382 | 133605393 |
| GCAAGGCGA | X | 135448906 | 135448929 |
| GGAACTTGA | 16 | 4523338 | 4523362 |
| GCAAGCAGA | 3 | 94928259 | 94928282 |
| GAAATTGAA | 1 | 66991943 | 66991956 |
| GAAATTTAA | 2 | 164605385 | 164605422 |
| GTAATTTGA | 7 | 148196702 | 148196715 |
| GAAATCTGA | 1 | 136087668 | 136087680 |
| GAAACTTGA | 1 | 94536891 | 94536904 |
