# Supplementary material for: Six Homeoproteins and a linc-RNA at the Fast MYH Locus Lock Fast Myofiber Terminal Phenotype
Source: PLoS Genet. 2014 May 22;10(5):e1004386. doi: 10.1371/journal.pgen.1004386 (PMC4031048; doi:10.1371/journal.pgen.1004386)
Supplement: Table S6 — MEF3 frequency matrix. (DOCX) [file pgen.1004386.s012.docx]

**Table S6.** MEF3 frequency matrix.

| A | 0.014 | 0.66 | 0.95 | 0.95 | 0.016 | 0.015 | 0.068 | 0.53 | 0.95 |
| --- | --- | --- | --- | --- | --- | --- | --- | --- | --- |
| C | 0.016 | 0.01 | 0.016 | 0.016 | 0.33 | 0.39 | 0.0092 | 0.013 | 0.016 |
| G | 0.95 | 0.0095 | 0.011 | 0.011 | 0.014 | 0.013 | 0.26 | 0.44 | 0.011 |
| T | 0.019 | 0.32 | 0.019 | 0.019 | 0.64 | 0.58 | 0.66 | 0.015 | 0.019 |
